# Polyketides from the Halotolerant Fungus *Myrothecium* sp. GS-17

**DOI:** 10.3390/molecules181215126

**Published:** 2013-12-06

**Authors:** Tao Liu, Jing Zhu, Song-Ya Zhang, Zhan-Lin Li, Li-Ping Guan, Hua-Qi Pan, Xin Wu, Jiao Bai, Hui-Ming Hua

**Affiliations:** 1 Department of Natural Products Chemistry, School of Pharmacy, China Medical University, Shenyang 110001, China; 2 State Key Laboratory of Magnetic Resonance and Atomic and Molecular Physics, Wuhan Institute of Physics and Mathematics, Chinese Academy of Sciences, Wuhan 430071, China; E-Mail: zhujing2008wd@163.com; 3 Key Laboratory of Structure-Based Drug Design & Discovery, Ministry of Education, School of Traditional Chinese Materia Medica, Shenyang Pharmaceutical University, Shenyang 110016, China; E-Mails: zsykk123@163.com (S.-Y.Z.); lzl1030@hotmail.com (Z.-L.L.); camellia58@sina.com (L.-P.G.); baijiao@hotmail.com (J.B.); huimhua@163.com (H.-M.H.); 4 Institute of Applied Ecology, Chinese Academy of Sciences, Shenyang 110016, China; E-Mail: panhq@iae.ac.cn; 5 School of Life Science and Biopharmaceutics, Shenyang Pharmaceutical University, Shenyang 110016, China; E-Mail: wuxin44771@163.com

**Keywords:** halotolerant fungus, *Myrothecium* sp., polyketide, furanone

## Abstract

Two new polyketides, myrothecol (**1**) and 5-hydroxy-3-methyl-4-(1- hydroxylethyl)-furan-2(5*H*)-one (**2**), were isolated from the fermentation broth of the halotolerant fungus *Myrothecium* sp. GS-17 along with three known compounds, 5-hydroxyl-3-[(1*S*)-1-hydroxyethyl]-4-methylfuran-2(5*H*)-one (**3**), 3,5-dimethyl-4- hydroxylmethyl-5-methoxyfuran-2(5*H*)-one (**4**), and 3,5-dimethyl-4-hydroxymethyl-5- hydroxyfuran-2(5*H*)-one (**5**). Compound **1** is the first natural occurring polyketide with a unique furylisobenzofuran skeleton. The structures of these compounds were established via extensive spectroscopic analyses including 1D-, 2D-NMR, HRESI-MS, and crystal X-ray diffraction analysis.

## 1. Introduction

The secondary metabolites of microorganisms that live under special circumstances have become an important source of pharmacologically active lead compounds. Halotolerant microorganisms have attracted the interest of many natural product chemists, due to their unique living environment and chemistry diversity [1,2,3,4]. In our search for novel antitumor compounds from halotolerant microorganisms, a halotolerant fungus named GS-17, which was authenticated as *Myrothecium* sp., was isolated from a soil sample taken from a saline field in Gansu Province in China. The fermentation broth of *Myrothecium* sp. GS-17 exhibited inhibitory activity against the human promyelocytic leukemia HL-60 cell line. Previous investigation of this fungus had led to the isolation of eight trichothecenes [5]. In our continuing study of the fungal metabolites of this organism, two new polyketides **1**, **2** and three known compounds **3**–**5** have now been isolated from its fermentation broth, and their structures were elucidated on the basis of extensive analyses of their spectroscopic evidence including 1D-, 2D-NMR, HRESI-MS and crystal X-ray diffraction. The paper deals with the isolation and structural elucidation of these compounds.

## 2. Results and Discussion

**Figure 1:**
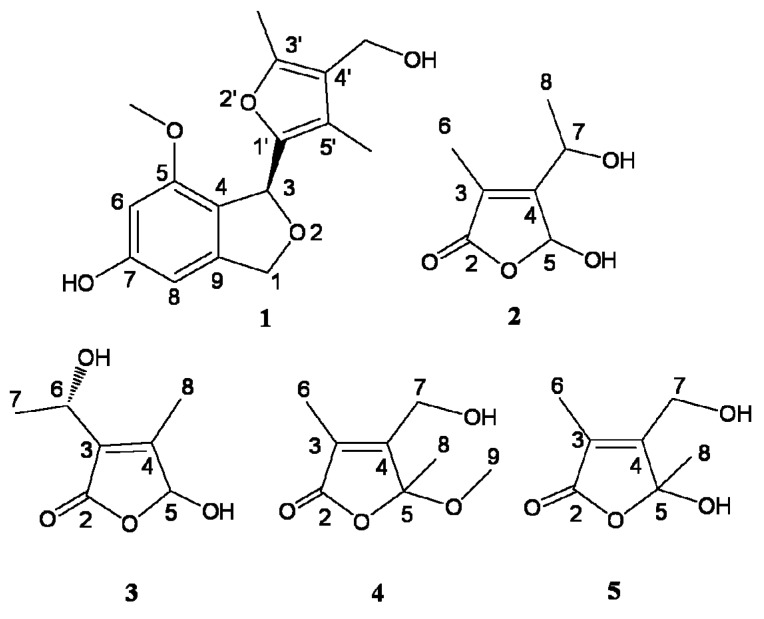
The structures of compounds **1**–**5**.

The extract of fermented broth of *Myrothecium* sp. GS-17 was fractionated by repeated column chromatography to yield five compounds **1**–**5** (Figure 1). Their structures were elucidated based on spectroscopic methods.

Compound **1** was obtained as colorless clustered crystals, and its molecular formula was determined as C_16_H_18_O_5_ on the basis of the HRESI-MS (*m/z* 291.1229, calcd. 291.1232 [M+H]^+^). The ^1^H-NMR (600 MHz, DMSO-*d*_6_) spectrum (Table 1) of **1** exhibited a pair of *meta*-coupled protons in an aromatic ring at *δ* 6.28 (1H, brs), and 6.25 (1H, brs). It also displayed the signals of one methoxyl at *δ* 3.59 (3H, s), two methyls at *δ* 2.10 (3H, s) and 1.80 (3H, s), and a phenolic hydroxyl at *δ* 9.51 (1H, s). In addition, it revealed the presence of an oxygenated methylene according to the protons at *δ* 4.97 (1H, dd, *J* = 2.5 Hz, 12.2 Hz), 4.83 (1H, d, *J* = 12.2 Hz), and a hydroxymethyl group according to the protons at *δ* 4.59 (1H, t, *J* = 5.3 Hz), 4.17 (2H, d, *J* = 5.3 Hz). The ^13^C-NMR spectrum (150 MHz, DMSO-*d_6_*) showed sixteen carbon signals including ten sp^2^ C-atoms at *δ* 159.5, 154.9, 146.9, 146.5, 142.4, 120.7, 117.3, 116.3, 99.6, and 98.0, indicating the presence of one aromatic ring and one furan ring. The ^1^H- and ^13^C-NMR data (Table 1) were assigned based on the HSQC and HMBC experiments.

**Table 1:** ^1^H- and ^13^C-NMR data of compound **1** (DMSO-*d*_6_, 600 and 150 MHz, resp., *δ* in ppm, *J* in Hz).

Position	δ(H)	δ(C)	Position	δ(H)	δ(C)
1	4.97, dd (12.2, 2.5)	72.1 (t)	1'		146.5 (s)
	4.83, d (12.2)				
3	6.02, d (2.5)	75.2 (d)	3'		146.9 (s)
4		116.3 (s)	4'		120.7 (s)
5		154.9 (s)	5'		117.3 (s)
6	6.25, brs	98.0 (d)	3'-CH_3_	2.10, s	11.6 (q)
7		159.5 (s)	4'-CH_2_OH	4.17, d (5.3)	53.2 (t)
8	6.28, brs	99.6 (d)	4'-CH_2_OH	4.59, t (5.3)	
9		142.4 (s)	5'-CH_3_	1.80, s	7.7 (q)
5-OCH_3_	3.59, s	55.3 (q)			
7-OH	9.51, s				

**Figure 2:**
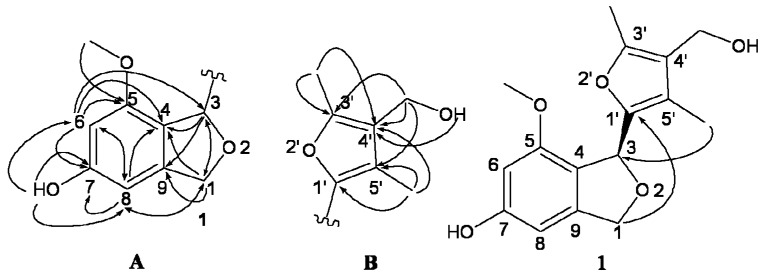
Key HMBC (H→C) correlations of compound **1**.

The HMBC spectrum showed the correlations of H-8 with C-1, C-6, C-4, C-7, of H-6 with C-8, C-4, C-5, C-7, of H-3 with C-1, C-4 and C-9, and of H-1 with C-3, C-4, C-8 and C-9, which revealed the presence of isobenzofuran fragment. The location of 7-OH and 5-OCH_3_ were determined by the HMBC correlations of 7-OH (*δ*_H_ 9.51) with C-6, C-7, C-8, and of 5-OCH_3_ (*δ*_H_ 3.59) with C-5. Based on the above evidence, the moiety A was elucidated as shown in Figure 2.

**Figure 3:**
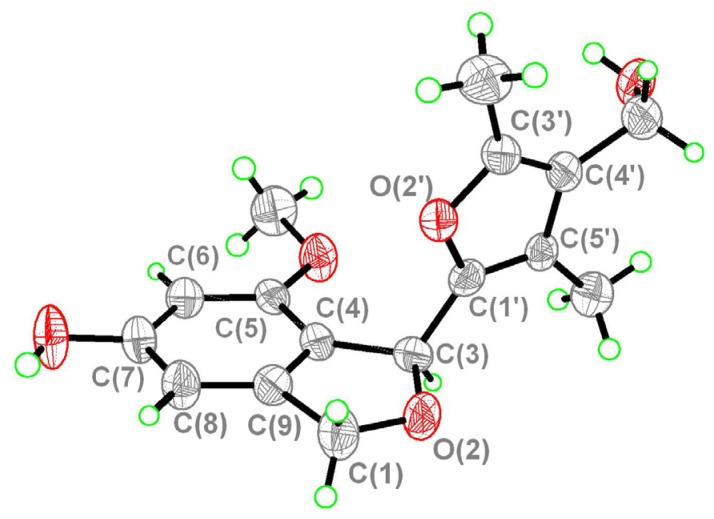
X-ray crystallographic diagram of compound **1**.

In the HMBC spectrum, the correlations of the methyl 5'-CH_3_ at *δ*_H_ 1.80 with C-5', C-4' and C-1', of 3'-CH_3_ at *δ*_H_ 2.10 with C-4' and C-3' and of 4'-CH_2_O- (*δ*_H_ 4.17) with C-5', C-4' and C-3' suggested the presence of 3',5'-dimethyl-4'-hydroxymethylfuran fragment (moiety B). In addition, the HMBC correlations of H-1 with C-1', and of 5'-CH_3_ (*δ*_H_ 1.80) with C-3, indicated that the moieties A and B linked together by C-3 and C-1' to form the structure as shown in Figure 2. X-ray crystallographic data further confirmed the structure of **1** to be as shown in Figure 3, and it was named as myrothecol. Due to the fact the crystallographic data collected by Mo Kα radiation, the absolute configuration was not determined.

Compound **2** was obtained as a colorless oil, and its molecular formula was determined as C_7_H_10_O_4_ on the basis of the HRESI-MS (*m/z* 181.0474, calcd. 181.0477 [M+Na]^+^). The IR spectrum indicated the presence of an *α*, *β*-unsaturated *γ*-lactone (1,752 cm^−1^). The ^1^H-NMR (300 MHz, DMSO-*d*_6_) spectrum (Table 2) of **2** revealed the presence of a methyl at *δ*_H_ 1.82 attached to a quaternary olefinic C-atom, a oxymethine at *δ*_H_ 6.13 (s, H-5) and a 1-hydroxyethyl at *δ*_H_ 1.29 (d, *J* = 6.7 Hz, H-8) and 4.62 (q, *J* = 6.7 Hz, H-7).

**Table 2:** ^1^H- and ^13^C-NMR data of compounds **2**–**5** (DMSO-*d*6, 300 and 75 MHz, resp., *δ* in ppm, *J* in Hz).

Position	2		3		4			5	
	δ(H)	δ(C)	δ(H)	δ(C)		δ(H)	δ(C)	δ(H)	δ(C)
2		172.4 (s)		170.5 (s)			171.5 (s)		171.5 (s)
3		123.8 (s)		131.0 (s)			128.1 (s)		123.7 (s)
4		162.0 (s)		157.6 (s)			156.6 (s)		160.4 (s)
5	6.13, s	97.9 (d)	6.05, s	97.9 (d)			107.8 (s)		107.3 (s)
6	1.82, s	8.6 (q)	4.52, q (6.6)	61.4 (d)		1.93, s	8.9 (q)	1.81, s	8.5 (q)
7	4.62, q (6.7)	62.9 (d)	1.26, d (6.6)	22.2 (q)		4.46, s	57.0 (t)	4.30, s	55.8 (t)
8	1.29, d (6.7)	21.9 (q)	2.01, s	11.3 (q)		1.63, s	22.9 (q)	1.52, s	24.8 (q)
MeO						3.21, s	51.2 (q)		
5-OH			7.71 brs						
6-OH			5.17 s						

**Figure 4:**
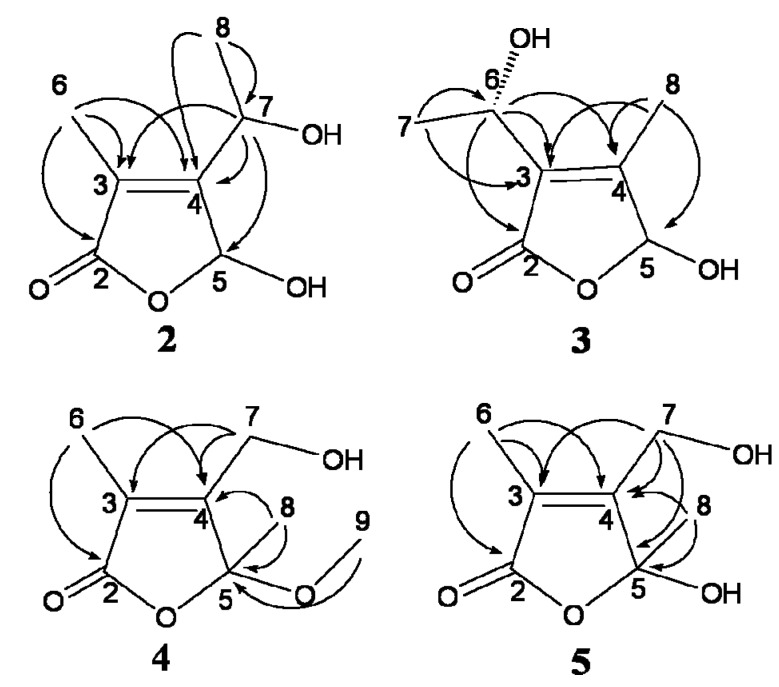
Key HMBC (H→C) correlations of compounds **2**–**5**.

The ^13^C-NMR (75 MHz, DMSO-*d*_6_) (Table 2) and HSQC spectra of **2** revealed seven C-atom signals, including one ester carbonyl group at *δ*_C_ 172.4, two olefinic C-atoms at *δ*_C_ 123.8 and 162.0, one hemiacetal C-atom at *δ*_C_ 97.9, one oxymethine at *δ*_C_ 62.9, and two methyl groups at *δ*_C_ 8.6 and 21.9. The above data suggested the presence of a furan-2(5*H*)-one skeleton. The methyl group was located at C-3 by the HMBC (Figure 4) correlations from H-6 (*δ*_H_ 1.82) to C-2 (*δ*_C_ 172.4), C-3 (*δ*_C_ 123.8) and C-4 (*δ*_C_ 162.0). The orientation of the 1-hydroxyethyl group was determined by the correlations from H-7 (*δ*_H_ 4.62) to C-3, C-4 and C-5 (*δ*_C_ 97.9), and from H-8 (*δ*_H_ 1.29) to C-7 (*δ*_C_ 62.9) and C-4. Thus, the structure of **2** was elucidated as 5-hydroxy-3-methyl-4-(1-hydroxyethyl)-furan-2(5*H*)-one.

Compound **3** was obtained as a colorless oil, and its molecular formula was determined as C_7_H_10_O_4_ on the basis of the HRESI-MS (*m/z* 181.0474, calcd. 181.0477 [M+Na]^+^). The absolute configuration at C-6 was deduced as *S* on the basis of its value of specific optical rotation ([α]^20^_D_ = −17.65) [6]. By comparison of its spectroscopic data with those reported previously, compound **3** was identified as 5-hydroxy-3-[(1*S*)-1-hydroxyethyl]-4-methylfuran-2(5*H*)-one [6].

Compound **4** was obtained as a colorless oil, and its molecular formula was determined as C_8_H_12_O_4_ on the basis of HRESI-MS (*m*/*z* 195.0623, calcd. 195.0633 [M+Na]^+^). Compound **4** was a racemate due to its value of specific optical rotation ([α]^20^_D_ = 0). By comparison of its spectroscopic data with those reported previously, the structure of **4** was elucidated as 3,5-dimethyl-4-hydroxymethyl-5-methoxy- furan-2(5*H*)-one [7].

Compound **5** was obtained as a colorless oil, and its molecular formula was determined as C_7_H_10_O_4_ on the basis of the HRESI-MS (*m*/*z* 181.0466, calcd. 181.0477 [M+Na]^+^). Compound **5** was also determined to be a racemate by its value of specific optical rotation ([α]^20^_D_ = 0). By comparison of its spectroscopic data with those reported previously, the structure of **5** was elucidated as 3,5-dimethyl-4-hydroxymethyl-5-hydroxyfuran-2(5*H*)-one [7].

## 3. Experimental

### 3.1. General Procedures

UV Spectra were measured on a UV-260 spectrophotometer (Shimadzu, Kyoto, Japan) IR (KBr) spectra were obtained on a Avatar 360-ESP spectrophotometer (Thermo Nicolet, Boston, MA, USA). ESI-MS Spectra were recorded on an Agilent 1100 ion trap spectrometer (Agilent Technologies, Palo Alto, CA, USA). HRESI-MS Spectra were recorded on a QFT-ESI mass spectrometer (Varian, Palo Alto, CA, USA). 1D- and 2D-NMR experiments were performed on Bruker ARX-300 and AV-600 NMR spectrometers (Bruker, Ettlingen, Germany) using DMSO-*d*_6_ as an internal standard. Optical rotations were measured on a Perkin-Elmer 241 polarimeter (Perkin-Elmer, Boston, MA, USA). RP-HPLC was carried out on a C_18_ column (10 mm × 250 mm, 10 μm; YMC, Kyoto, Japan) on a Hitachi (Tokyo, Japan) L-2000 HPLC system equipped with a Hitachi L2400 UV-detector. Silica gel (100–200, 200–300 mesh, Qingdao Ocean Chemical Co., Qingdao, China), ODS (50 µm, YMC) and Sephadex LH-20 (GE Healthcare, Uppsala, Sweden) for column chromatography as well as silica gel GF_254_ (10–40 μm, Qingdao Ocean Chemical Co) for TLC were used.

### 3.2. Fungal Material and Culture

The halotolerant fungus *Myrothecium* sp. GS-17 was obtained from the soil samples obtained from a saline field located in Gansu Province in China. On the basis of its macroscopic appearance and 18S rDNA gene sequence, GS-17 was identified as *Myrothecium* sp., and preserved in Department of Natural Products Chemistry, Shenyang Pharmaceutical University, P. R. China. A small loop of spores growing on a Martin slant was inoculated into a 250 mL Erlenmeyer flask containing 75 mL sea-water-based culture medium (maltose 2%, monosodium glutamate 1%, KH_2_PO_4_ 0.05%, MgSO_4_·7H_2_O 0.03%, glucose 1%, yeast extract 0.3%, corn steep liquor 0.1%, mannitol 2%, sea water, pH 6.5) and cultured at 28 °C for 2 days on a rotary shaker at 180 r·min^−1^. Then, 10 mL of the resultant seed culture was inoculated into a 500 mL Erlenmeyer flask containing the above culture medium (150 mL) and incubated (500 flasks) for 8 days under the same conditions.

### 3.3. Extraction and Isolation

The fermented broth (56 L) was filtered through cheesecloth to yield the supernatant and mycelia. The supernatant was subjected to HPD100 macroporous absorption resin chromatography, eluted in gradient with EtOH/H_2_O 30%, 70%, 100% to yield three fractions (Frs*.* 1–3). Fr. 2 (20 g) was fractioned on a silica gel column eluting with CH_2_Cl_2_–MeOH (from 100:0 to 0:100) to afford sixty fractions (SubFrs. 1–60). SubFr. 2 was further separated by a flash silica gel column eluting with petroleum ether–acetone (from 11:1 to 3:1) to give three fractions (SubFrs*.* 2A–2C). SubFr*.* 2A was further purified by RP-HPLC (MeOH–H_2_O: from 10% to 30%) to yield compounds **2** (7.0 mg), **3** (8.1 mg), **4** (14.2 mg) and **5** (10.0 mg). SubFr*.* 29 was purified by recrystallization from methanol to yield **1** (5.8 mg).

#### 3.3.1. Myrothecol (**1**)

Colorless clustered crystals. IR (KBr) *ν*_max_: 3332, 3154, 1609, 1477, 1117 cm^−1^. ^1^H-NMR (600 MHz, DMSO-*d*_6_) and ^13^C-NMR (150 MHz, DMSO-*d*_6_): see Table 1. HRESI-MS: *m/z* 291.1229 ([M+H]^+^, C_16_H_19_O_5_; calcd. 291.1232).

#### 3.3.2. 5-Hydroxy-3-methyl-4-(1-hydroxyethyl)-furan-2(5*H*)-one (**2**)

Colorless oil. [α]^20^_D_ = –7.53 (*c* 0.11, MeOH). UV (MeOH) *λ*_max_: 208 nm. IR (KBr) *ν*_max_: 3425, 1752, 1052, 1027, 1006 cm^−1^. ^1^H-NMR (300 MHz, DMSO-*d*_6_) and ^13^C-NMR (75 MHz, DMSO-*d*_6_): see Table 2. ESI-MS: 156.9 [M–H]^−^. HRESI-MS: 181.0474 ([M+Na]^+^, C_7_H_10_NaO_4_^+^; calcd. 181.0477).

#### 3.3.3. 5-Hydroxy-3-[(1*S*)-1-hydroxyethyl]-4-methylfuran-2(5*H*)-one (**3**)

Colorless oil. [α]^20^_D_ = –17.65 (*c* 0.07, MeOH). UV (MeOH) *λ*_max_: 205 nm. IR (KBr) *ν*_max_: 3401, 2983, 1749, 1050, 1029 cm^−1^. ^1^H-NMR (300 MHz, DMSO-*d*_6_) and ^13^C-NMR (75 MHz, DMSO-*d*_6_): see Table 2. ESI-MS: 156.9 [M–H]^−^. HRESI-MS: 181.0474 ([M+Na]^+^, C_7_H_10_NaO_4_^+^; calcd. 181.0477).

#### 3.3.4. 3,5-Dimethyl-4-hydroxymethyl-5-methoxyfuran-2(5*H*)-one (**4**)

Colorless oil. [α]^20^_D_ = 0 (*c* 0.20, MeOH). UV (MeOH) *λ*_max_: 210 nm. IR (KBr) *ν*_max_: 3399, 2938, 1758, 1071, 1026 cm^−1^. ^1^H-NMR (300 MHz, DMSO-*d*_6_) and ^13^C-NMR (75 MHz, DMSO-*d*_6_): see Table 2. ESI-MS: 173.1 [M+H]^+^. HRESI-MS: 195.0623 ([M+Na]^+^, C_8_H_12_NaO_4_^+^; calcd. 195.0633).

#### 3.3.5. 3,5-Dimethyl-4-hydroxymethyl-5-hydroxyfuran-2(5*H*)-one (**5**)

Colorless oil. [α]^20^_D_ = 0 (*c* = 0.50, MeOH). UV (MeOH) *λ*_max_: 207 nm. IR (KBr) *ν*_max_:3368, 2993, 2933, 1749, 1199, 1044 cm^−1^. ^1^H-NMR (300 MHz, DMSO-*d*_6_) and ^13^C-NMR (75 MHz, DMSO-*d*_6_): see Table 2. ESI-MS: 156.9 [M–H]^−^. HRESI-MS: 181.0466 ([M+Na]^+^, C_7_H_10_NaO_4_^+^; calcd. 181.0477). 

### 3.4. Single Crystal X-ray Structure Determination of Myrothecol (**1**)

The crystal structure of **1** was determined using data collected on a SMART APEX II CCD diffractometer (Mo Kα radiation). A suitable colorless crystal (0.18 × 0.14 × 0.12 mm) of **1** for diffraction was obtained from methanol solution. Crystal data: C_16_H_18_O_5_ orthorhombic, a = 7.180(5) Å, b = 9.723(5) Å, c = 10.924(5) Å, *V* = 722.6(7) Å^3^, *Z* = 2, D_calcd_ 1.334 mg/m^3^, λ = 0.71069 Å, *F*(000) = 308, *T* = 293(2) K. A total of 2433 reflections were collected, of which 1545 unique reflections (R_int_ = 0.0532) with I > 2σ (*I*) were used for the analysis. The data was solved using direct method SHELXL-97, and the structure was refined full-matrix least-squares on *F*^2^ values. The refined structure model converged to a final *R*1 0.0476, *wR*2 = 0.1205 with Goodness-of-fit = 0.951. CCDC 969517 contains the supplementary crystallographic data for **1**. These data can be obtained free of charge via http://www.ccdc.cam.ac.uk/conts/retrieving.html (or from the CCDC, 12 Union Road, Cambridge CB2 1EZ, UK; Fax: +44 (0) 1223 336033; E-mail: deposit@ccdc.cam.ac.uk).

## 4. Conclusions

Our investigation on the metabolites of the halotolerant fungus *Myrothecium* sp. GS-17 has resulted in the isolation of five polyketides, including two new ones, myrothecol and 5-hydroxy-3-methyl- 4-(1-hydroxyethyl)-furan-2(5*H*)-one. Myrothecol (**1**) represents a novel class of natural polyketides.

## Supplementary Materials

Supplementary materials can be accessed at: http://www.mdpi.com/1420-3049/18/12/15126/s1.

## References

[1] Wang W.L., Lu Z.Y., Tao H.W., Zhu T.J., Fang Y.C., Gu Q.Q., Zhu W.M. (2007). Isoechinulin-type alkaloids, variecolorins A–L, from halotolerant *Aspergillu. variecolor*. J. Nat. Prod..

[2] Socha A.M., Long R.A., Rowley D.C. (2007). Bacillamides from a hypersaline microbial mat bacterium. J. Nat. Prod..

[3] Caton T.M., Witte L.R., Ngyuen H.D., Buchheim J.A., Buchheim M.A., Schneegurt M.A. (2004). Halotolerant aerobic heterotrophic bacteria from the great salt plains of Oklahoma. Microb. Ecol..

[4] Ventosa A., Nieto J.J., Oren A. (1998). Biology of moderately halophilic aerobic bacteria. Microbiol. Mol. Biol. Rev..

[5] Zhang S.Y., Li Z.L., Guan L.P., Wu X., Pan H.Q., Bai J., Hua H.M. (2012). Structure determination of two new trichothecenes from a halotolerant fungus *Myrothecium.* sp. GS-17 by NMR spectroscopy. Mag. Reson. Chem..

[6] Grossmann G., Poncioni M., Bornand M., Jolivet B., Neuburger M., Séquin U. (2003). Bioactive butenolides from *Streptomyces. Antibioticus* TÜ 99: Absolute configurations and synthesis of analogs. Tetrahedron.

[7] Xu H., Jiang N., Guo Y., Jiao R.H., Cui J.T., Song Y.C., Tan R.X. (2011). Fully substituted unsaturated lactones from endophytic *Myrothecium*. sp. J. Asian. Nat. Prod. Res..

