# Supplementary material for: Polyketides from the Halotolerant Fungus Myrothecium sp. GS-17
Source: Molecules. 2013 Dec 6;18(12):15126–33. doi: 10.3390/molecules181215126 (PMC6270343; doi:10.3390/molecules181215126)
Supplement: Supplementary file 1 [file molecules-18-15126-s001.pdf]

Supplementary Materials

Figure S1. The ^{1}H-NMR Spectrum of Compound **1** in DMSO-d_6.

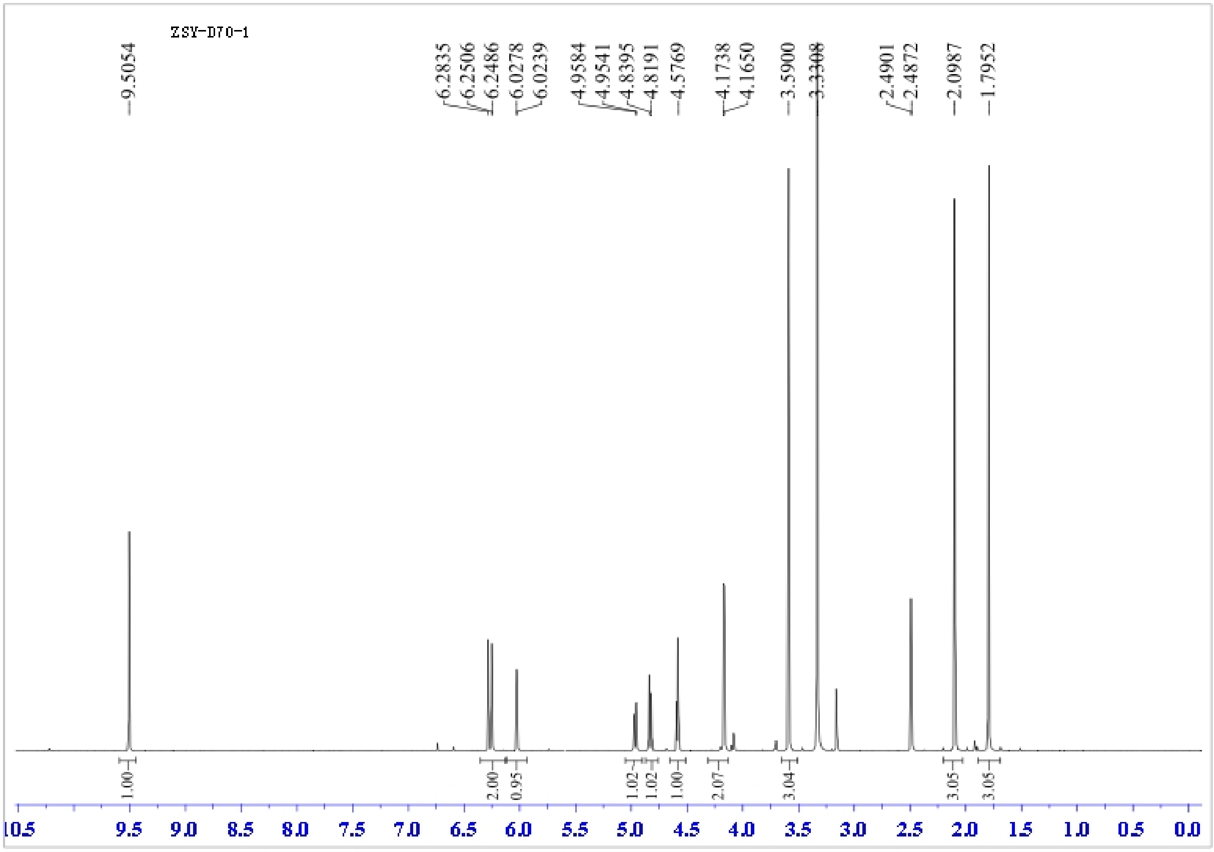

Figure S2. The ^{13}C-NMR Spectrum of Compound **1** in DMSO-d_6.

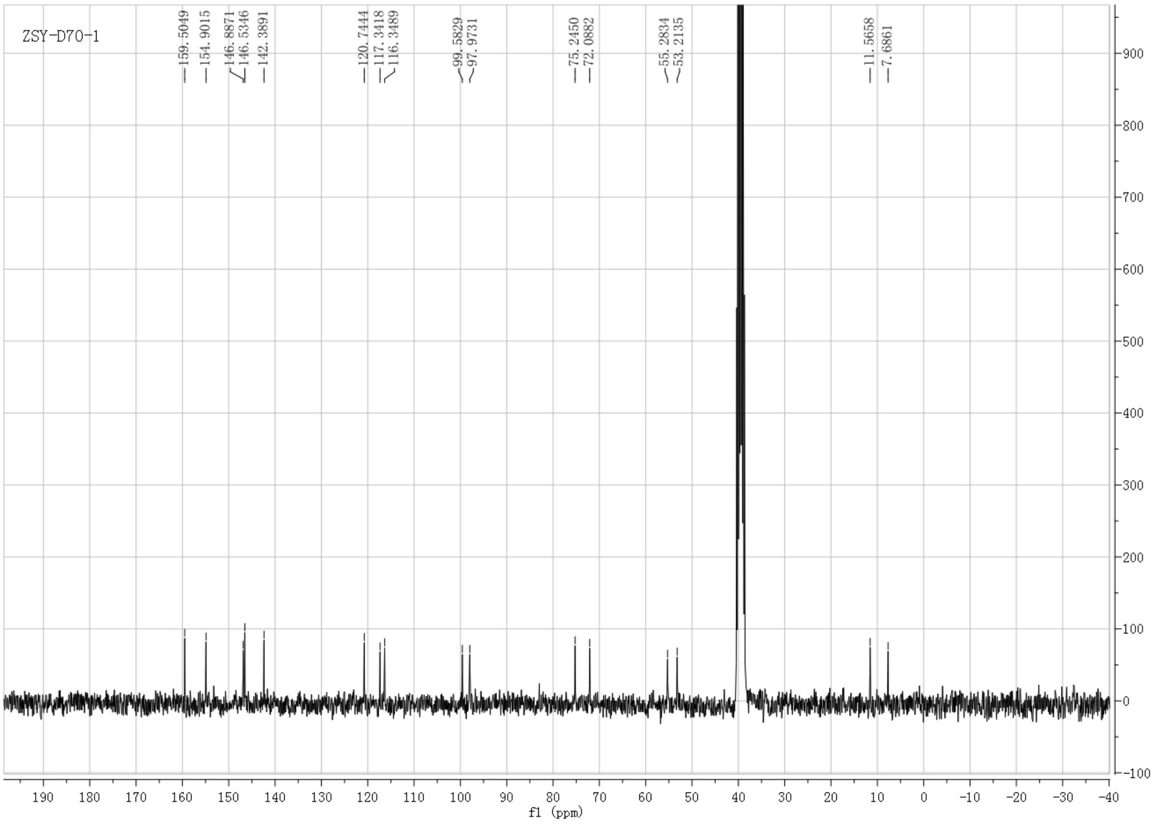

Figure S3. The HSQC Spectrum of Compound **1** in DMSO-d_6.

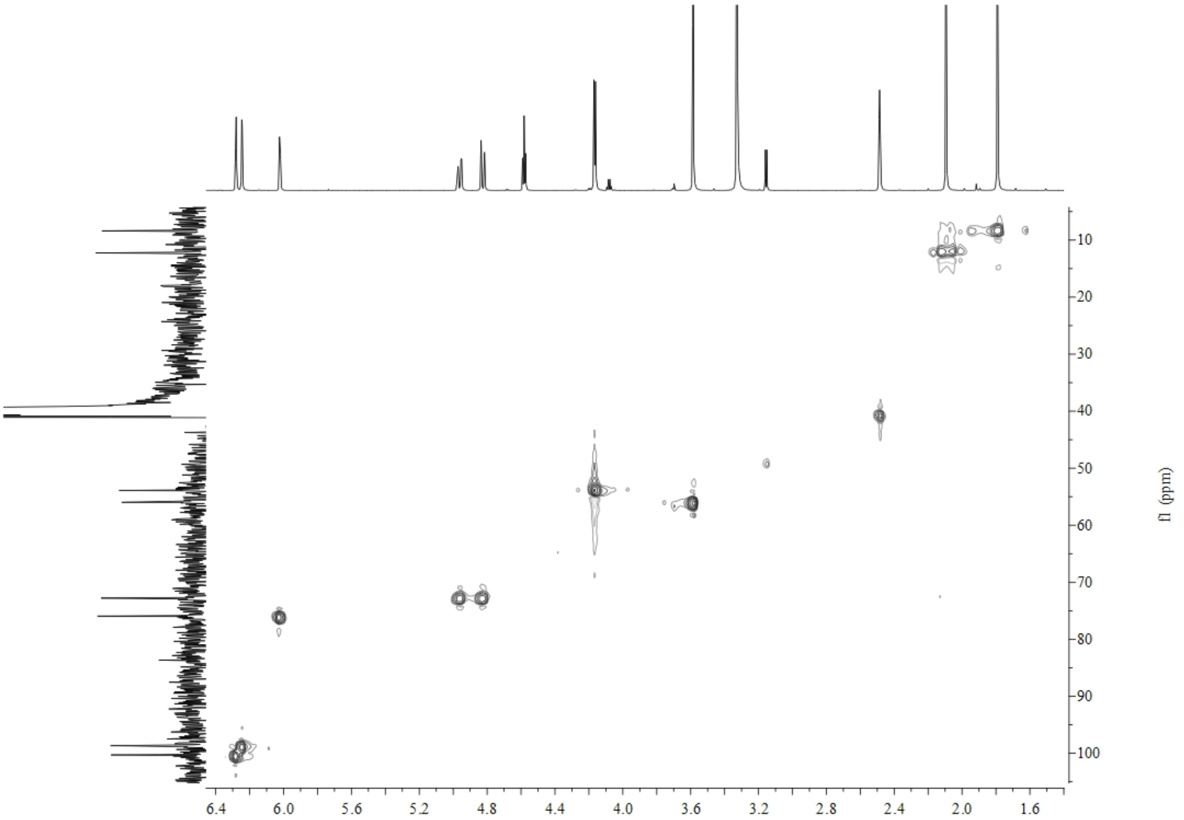

Figure S4. The HMBC Spectrum of Compound **1** in DMSO-d_6.

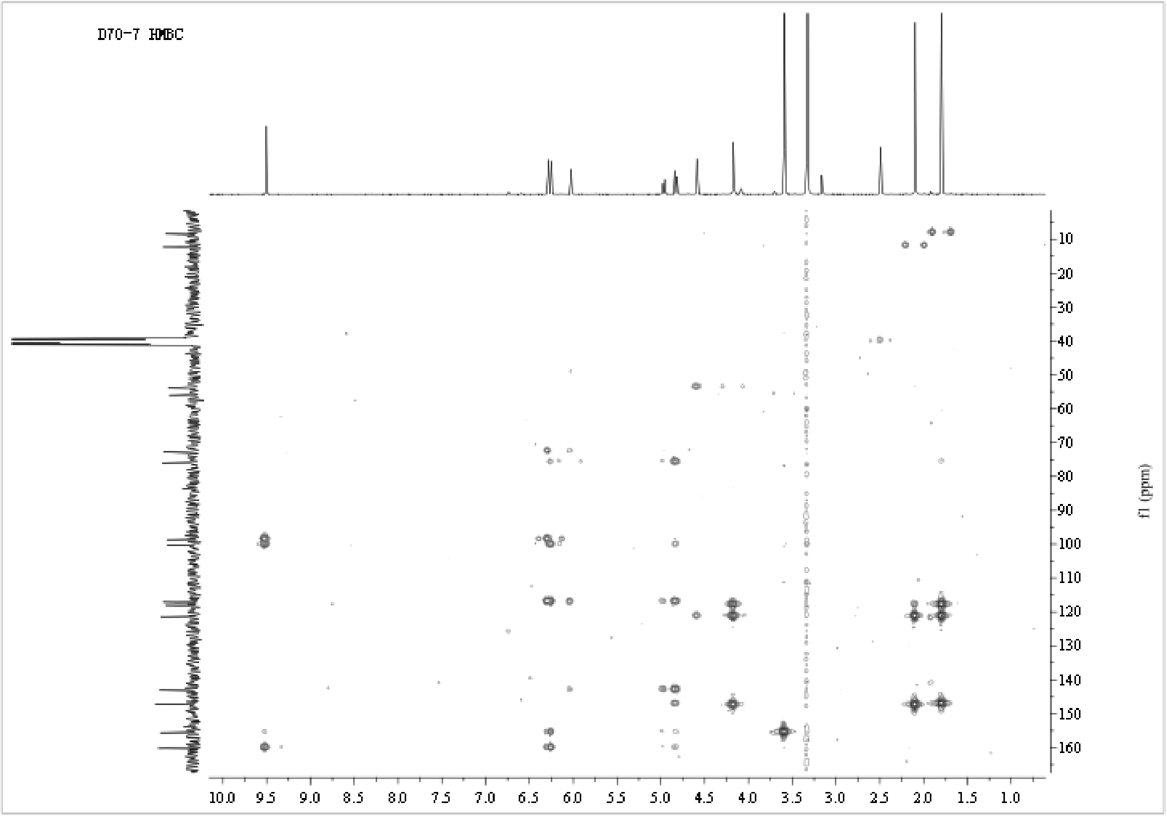

Figure S5. The ^{1}H-^{1}H COSY Spectrum of Compound **1** in DMSO-d_6.

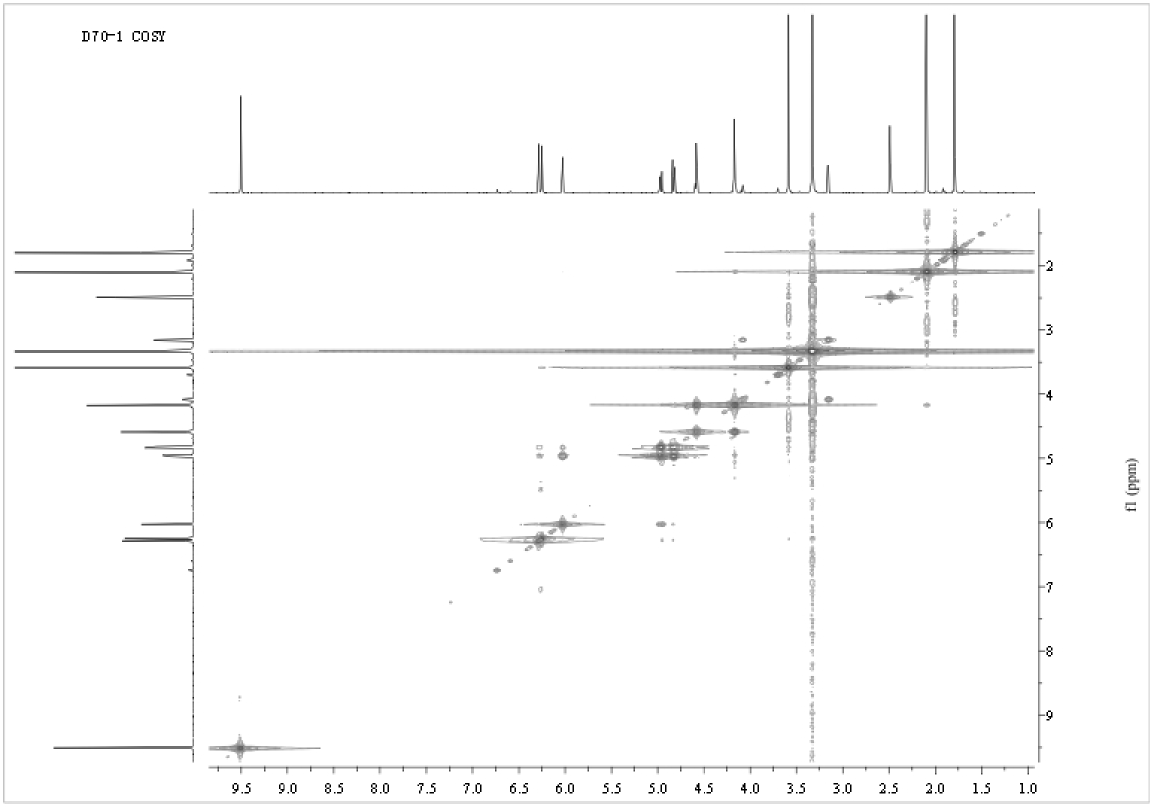

Figure S6. The NOESY Spectrum of Compound **1** in DMSO-d_6.

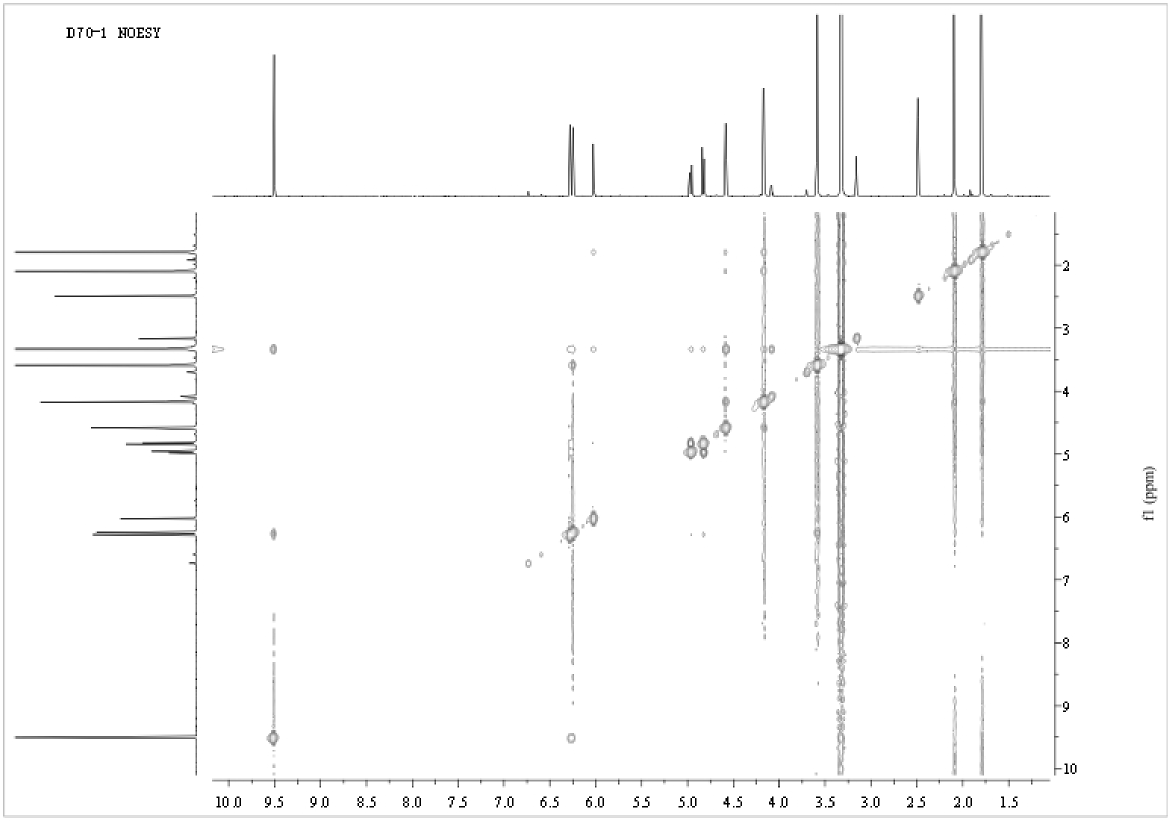

Figure S7. The IR Spectrum of Compound **1**.

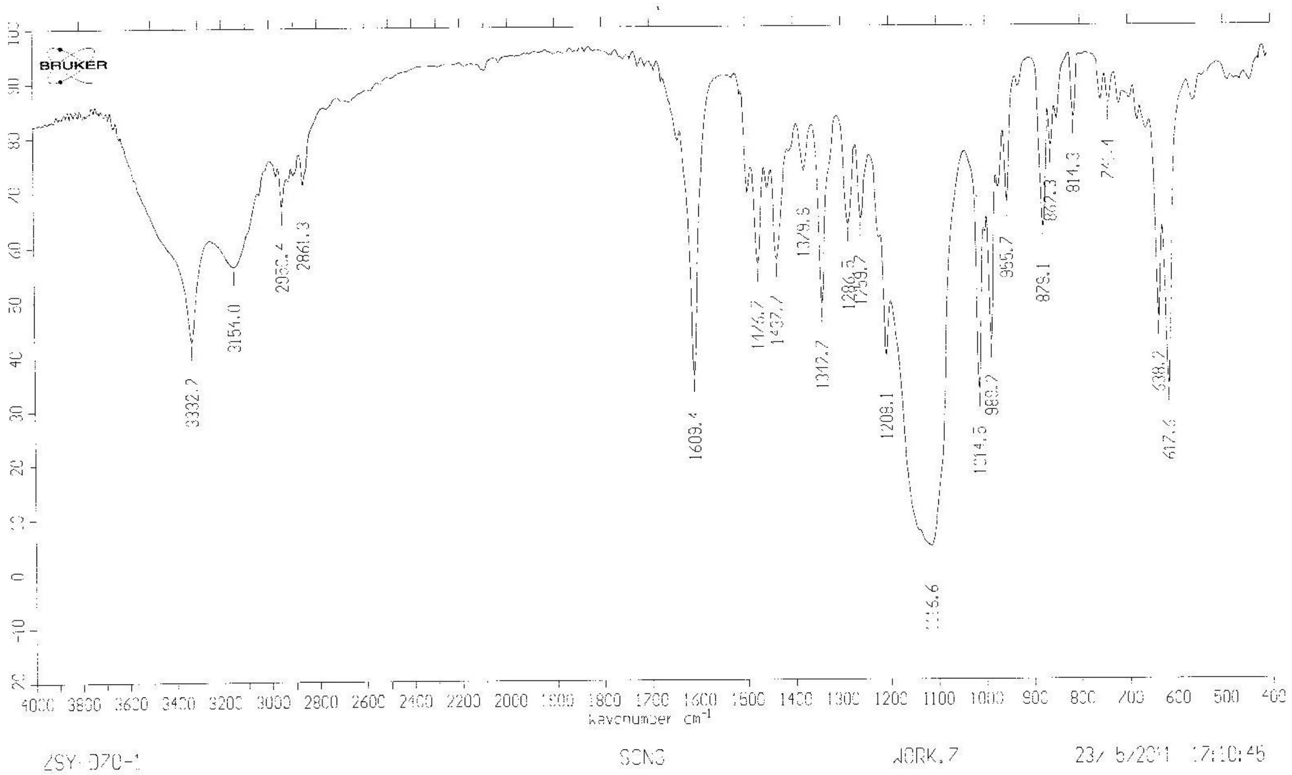

Figure S8. The HRESI-MS Spectrum of Compound **1**.

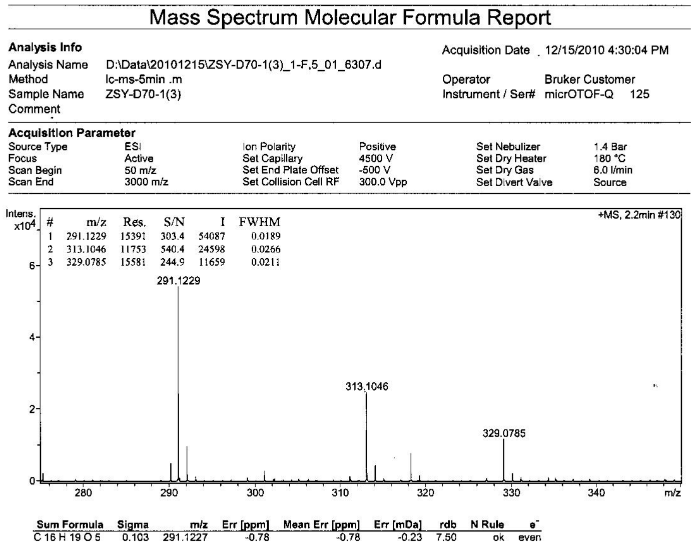

Bruker Daltonics DataAnalysis 3.4 printed: 12/16/2010 3:56:34 PM Page 1 of 1

Figure S9. The ^{1}H-NMR Spectrum of Compound **2** in DMSO-d_6.

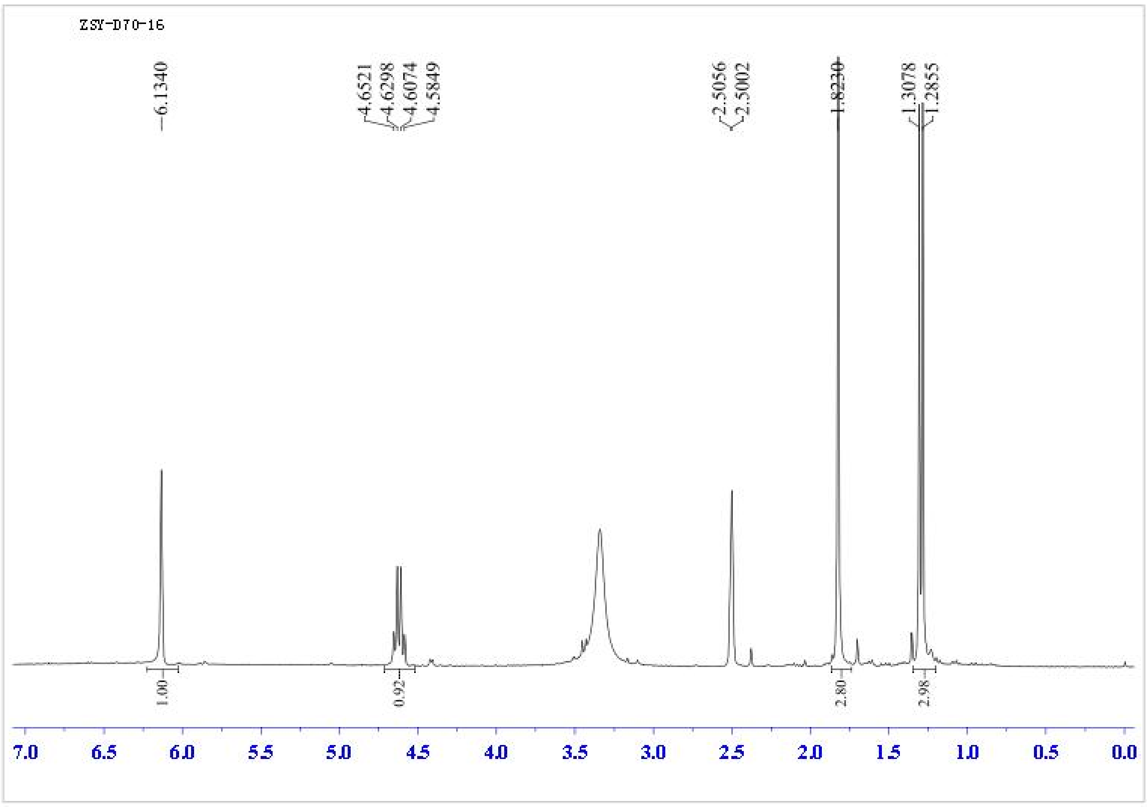

Figure S10. The ^{13}C-NMR Spectrum of Compound **2** in DMSO-d_6.

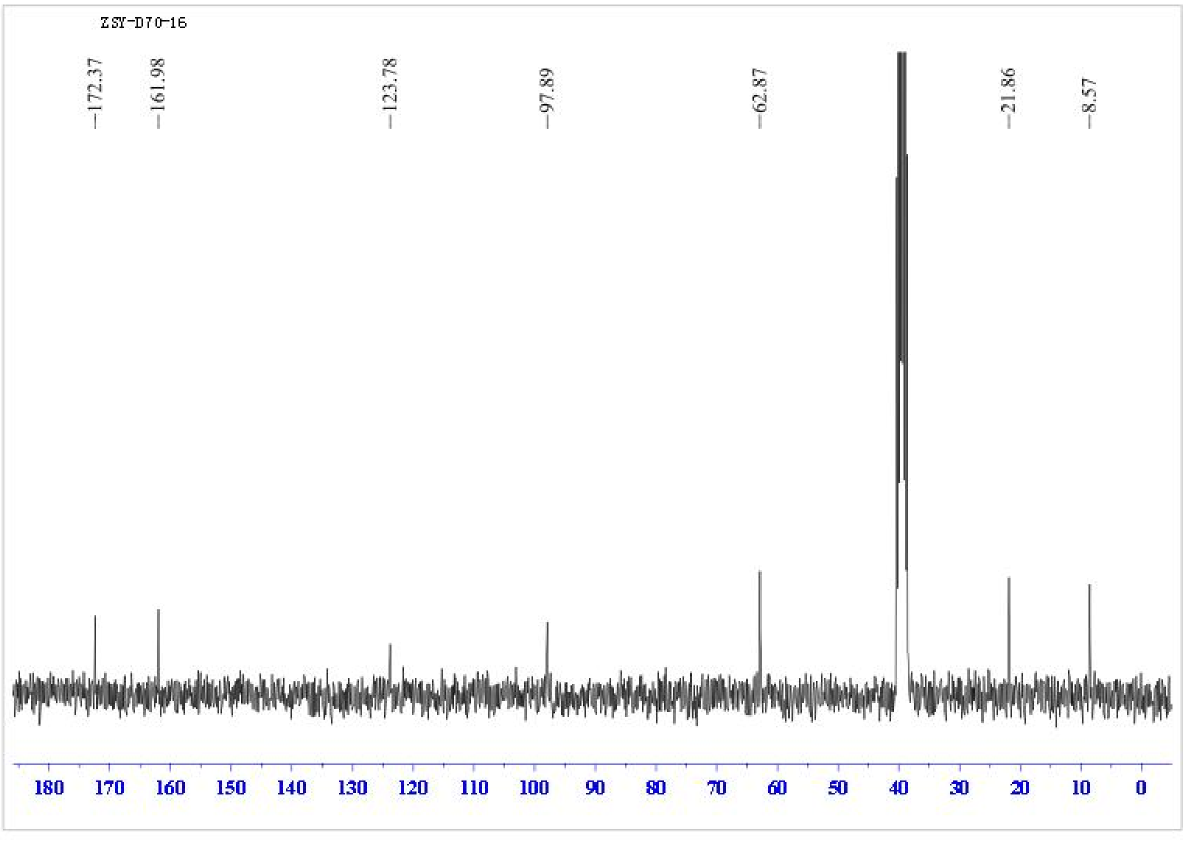

Figure S11. The HSQC Spectrum of Compound **2** in DMSO-d_6.

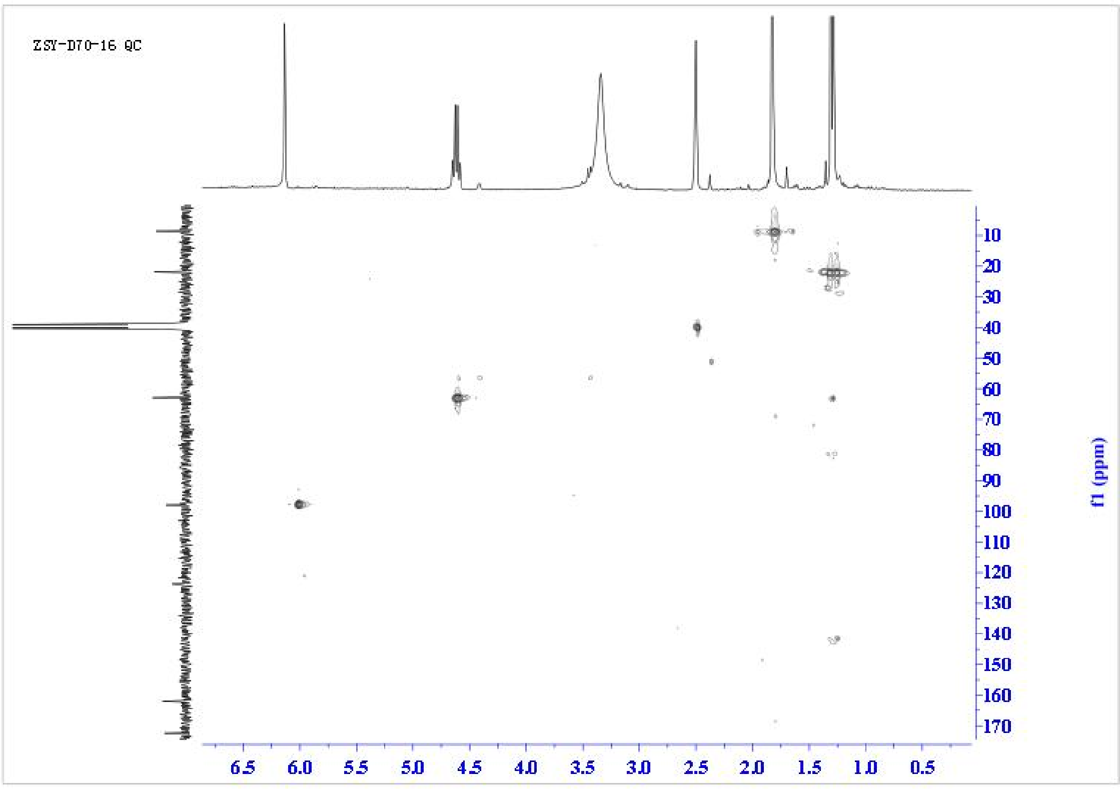

Figure S12. The HMBC Spectrum of Compound **2** in DMSO-d_6.

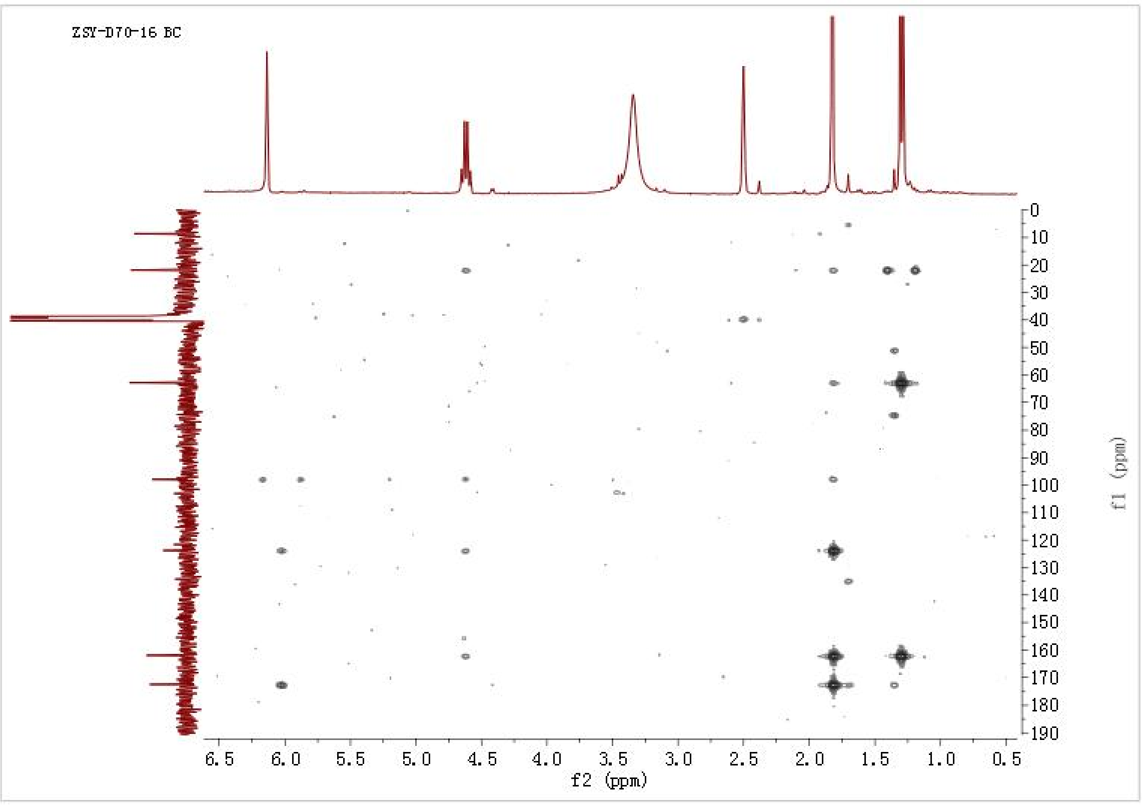

Figure S13. The IR Spectrum of Compound **2**.

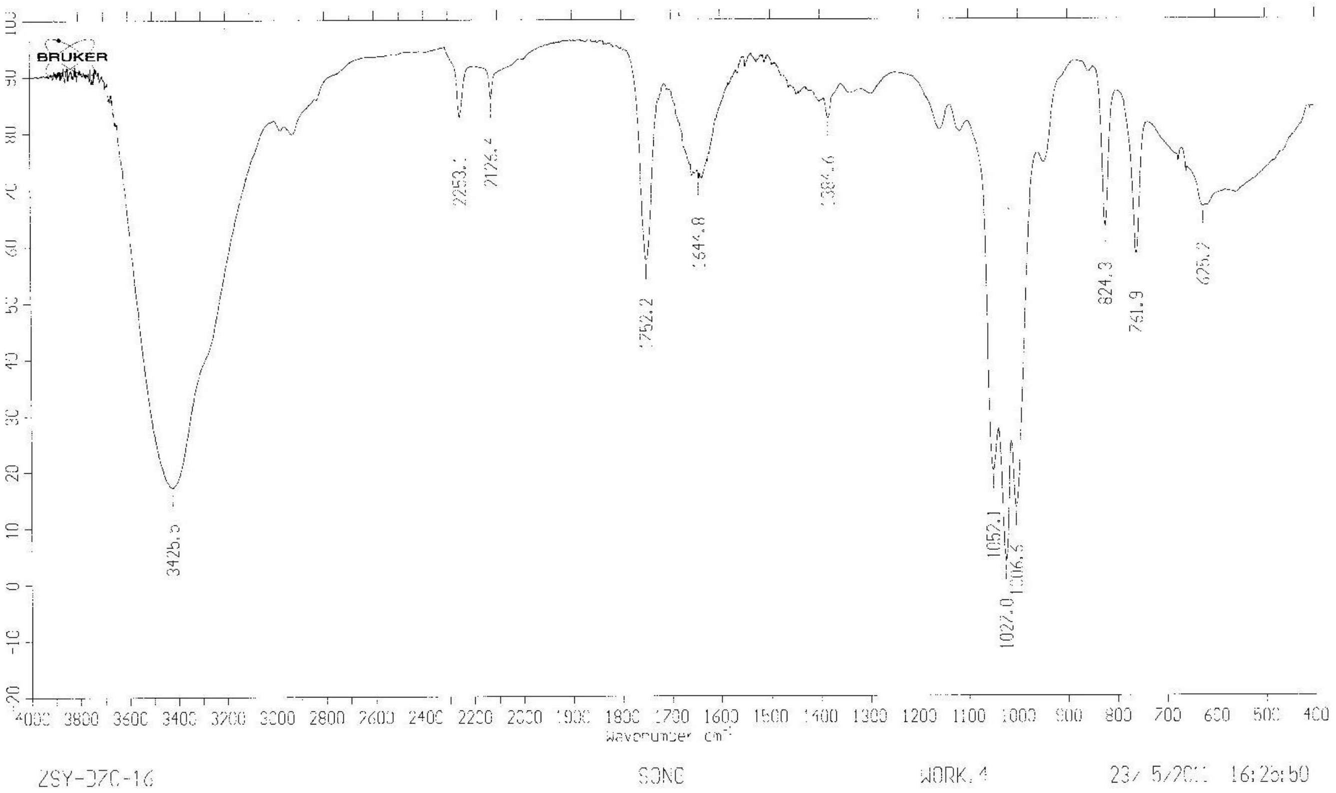

Figure S14. The UV Spectrum of Compound **2** in MeOH.

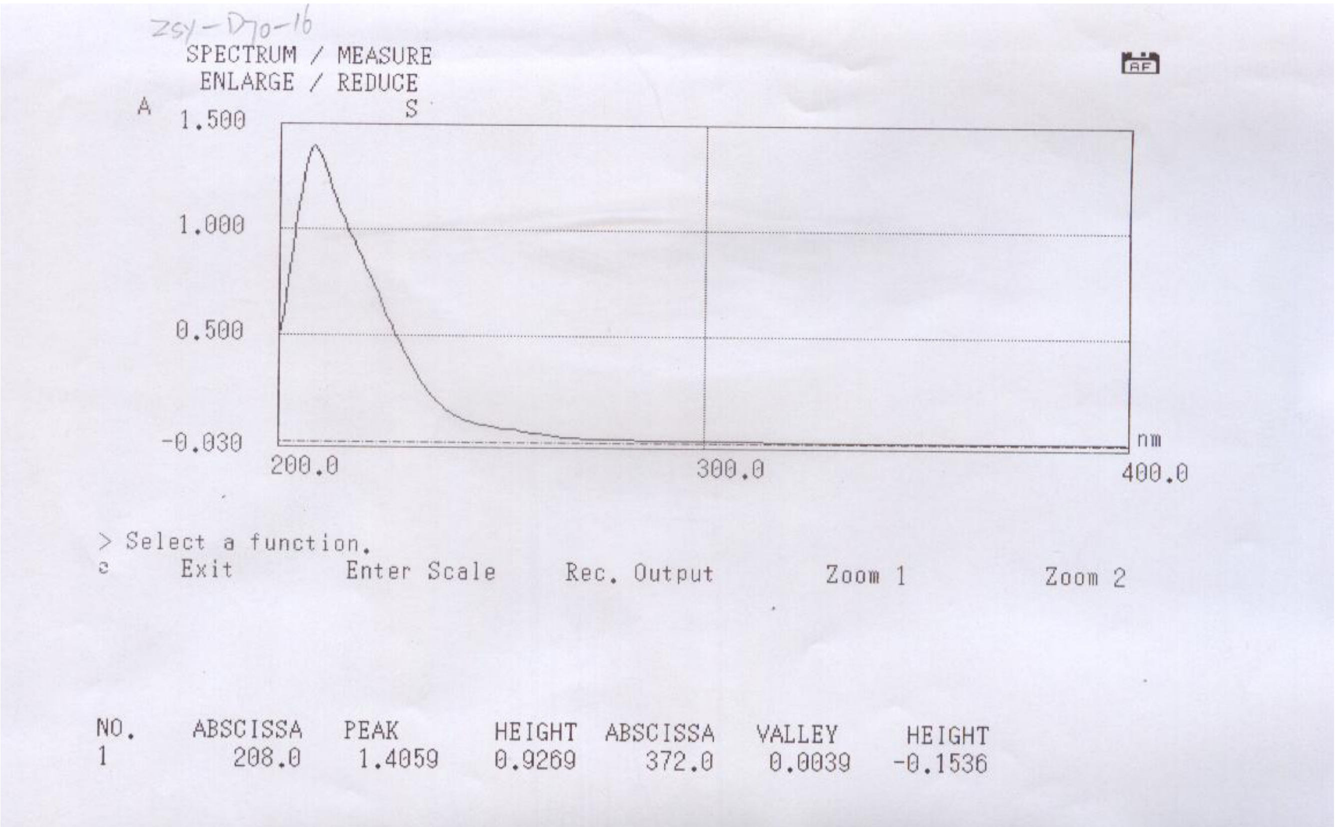

Figure S15. The ESI-MS Spectrum of Compound **2**.

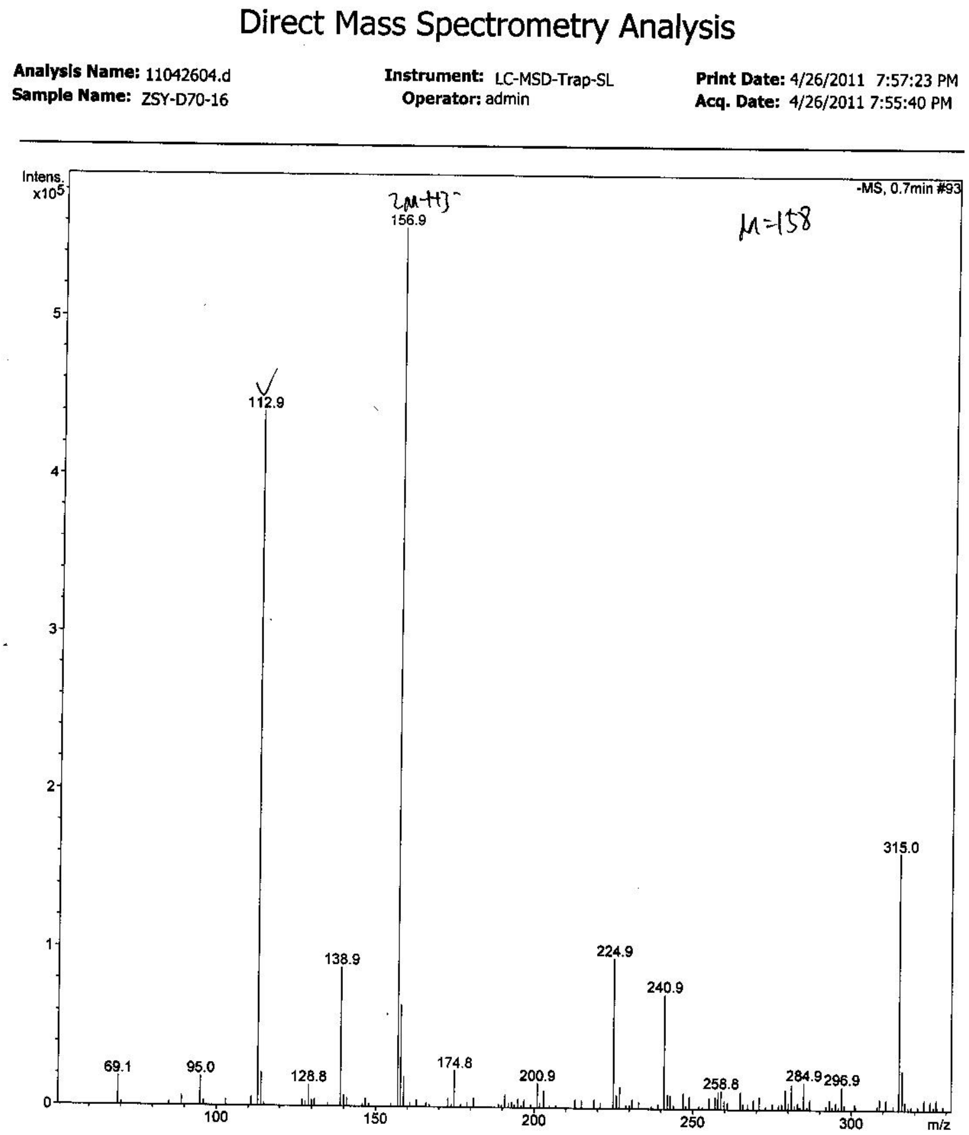

Figure S16. The HRESI-MS Spectrum of Compound **2**.

Mass Spectrum Molecular Formula Report

Analysis Info

Analysis Name	D:\Data\20110516\zsy-d70-16.d	Acquisition Date	5/16/2011 4:06:44 PM
Method	tune_50-1000_POS_20110328.m	Operator	YP
Sample Name	zsy-d70-16	Instrument / Ser#	micrOTOF-Q 125
Comment			

Acquisition Parameter

Source Type	ESI	Ion Polarity	Positive	Set Nebulizer	0.5 Bar
Focus	Active	Set Capillary	4500 V	Set Dry Heater	180 °C
Scan Begin	50 m/z	Set End Plate Offset	-500 V	Set Dry Gas	4.0 l/min
Scan End	3000 m/z	Set Collision Cell RF	35.0 Vpp	Set Divert Valve	Source

Generate Molecular Formula Parameter

Formula, min.	C7H10Na1O4				
Formula, max.					
Measured m/z	181.047	Tolerance	4 mDa	Charge	1
Check Valence	no	Minimum	0	Maximum	0
Nirogen Rule	no	Electron Configuration	both		
Filter H/C Ratio	no	Minimum	0	Maximum	3
Estimate Carbon	yes				

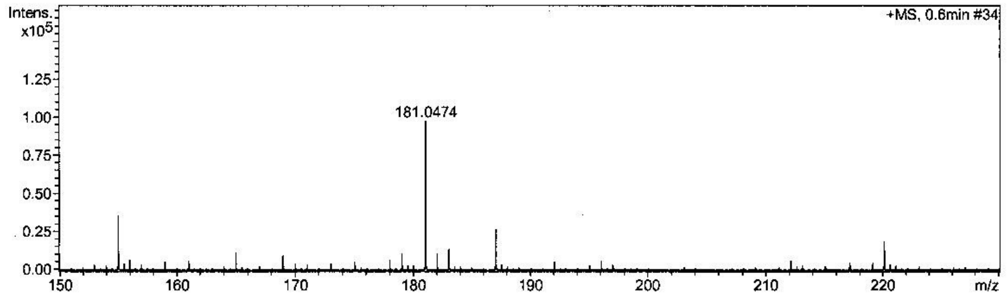

#	m/z	I
1	181.0474	97596

Sum Formula	Sigma	m/z	Err [ppm]	Mean Err [ppm]	Err [mDa]	rdb	N Rule	e⁻
C 7 H 10 Na 1 O 4	0.075	181.0471	-1.74	-3.16	-0.31	2.50	ok	even
